# Sperm vitrification *of Prochilodus brevis*: influence of diluent, stored volume and supplementation with sulfated polysaccharides of Nile tilapia (*Oreochromis niloticus*) skin

**DOI:** 10.1590/1984-3143-AR2024-0075

**Published:** 2025-02-07

**Authors:** Priscila Silva de Almeida-Monteiro, Romulo Roberto Ribeiro Pinheiro, Mayara Setúbal Oliveira-Araújo, Thais Maia Torres, Renata Vieira do Nascimento, Vanessa Alves Pereira, Yasmim Maia Ferreira, Yara Silvino Sales, Jéssica Sales Lobato, Ianna Wivianne Fernandes Araújo, José Ariévilo Gurgel Rodrigues, Assis Rubens Montenegro, Carminda Sandra Brito Salmito-Vanderley

**Affiliations:** 1 Programa de Pós-Graduação em Ciências Veterinárias, Faculdade de Veterinária, Universidade Estadual do Ceará, Fortaleza, CE, Brasil; 2 Universidade Federal do Ceará, Fortaleza, CE, Brasil

**Keywords:** cryopreservation, fast freezing, sperm conservation, freezing medium, powder coconut water (ACP-104), stored volume, glycosaminoglycans

## Abstract

The aim of this study was to evaluate the influence of diluent, stored volume and the cryodiluent medium supplementation with sulfated polysaccharides (SP) extracted from Nile tilapia skin on *P. brevis* vitrified sperm. Six pools were diluted in 5% Glucose or Powder Coconut Water (ACP-104), supplemented or not with 0.5 mg/mL of SP, and submitted to vitrification. Subsequently, they were stored in cryotubes in two volumes (60 μL and 420 μL). After 15 days, the samples were devitrified and evaluated for kinetics, membrane integrity and sperm DNA integrity. ACP-104 proved to be the best diluent for *P. brevis* sperm vitrification. Membrane and DNA integrity rates were higher (P < 0.05) when stored in smaller and larger volume, respectively. Additionally, the best rates (P < 0.05) of these same parameters were obtained with supplemented medium. There was interaction (P < 0.05) between diluent and stored volume, with ACP-104 exceeding 5% Glucose for motility in both volumes, while for average path speed (VAP) and membrane integrity the same happened in the larger volume. 5% Glucose had higher VAP and membrane integrity when stored in smaller volume. There was a triple interaction (P < 0.05) for DNA integrity, and better results were obtained when semen was vitrified in ACP-104 and stored in the larger volume, regardless of supplementation, which influenced only the 5% Glucose medium in the smaller volume. It was concluded that ACP-104, supplemented with SP and stored in larger volume make up the best treatment for *P. brevis* sperm vitrification.

## Introduction

Semen cryopreservation is a promising reproductive biotechnology for the development of aquaculture, as it can facilitate the production and captive breeding, especially of rheophilic species, through artificial fertilization. The technique consists of freezing male gametes in liquid nitrogen (-196 °C), thus keeping them viable, with preserved structure and functionality, indefinitely (Bakhach, 2009). Cryopreservation procedures can be performed slowly (conventional method) or quickly, a technique known as vitrification (Figueroa et al., 2015).

The vitrification involves immersing the sample directly into liquid nitrogen, without the need for equilibrium time or sophisticated equipment. Its main advantage is the significant reduction in ice crystal formation, as the sample reaches a viscous and amorphous state known as the vitreous state (Naik et al., 2005; Rubinsky, 2003). To achieve this state, the sample must be frozen in small volumes to increase freezing/thawing rates (Xin et al., 2017). However, storing samples in small volumes may not be feasible for large-scale production, as an ideal ratio of spermatozoa of oocyte is required to ensure the success of artificial fertilization, a parameter known as inseminating dose (Oliveira-Araújo et al., 2020). Thus, it is necessary to conduct studies that test different stored volumes to define an ideal one that ensures a good post-thawing quality, and then could be used in the routine fertilization.

Additionally, for successful cryopreservation in general, it’s necessary to dilute the semen sample in a freezing medium, composed mainly of a diluent and a cryoprotectant (Salmito-Vanderley et al., 2012). The effectiveness of these components depends on the species to which it is being applied (Almeida-Monteiro et al., 2020a) necessitating testing of the most suitable solutions.

Glucose stands out as one of the most widely used diluents for conventional cryopreservation of fish semen. However, for vitrification, more complex diluents are applied, such as *Belltsville Thawing Solution* (BTS) (Varela-Junior et al., 2015), Medium Cortland^®^ (Merino et al., 2011; Figueroa et al., 2013, 2015), modified medium Tanaka (Kása et al., 2017) and Powder Coconut Water specific for fish (ACP-104) (Almeida-Monteiro et al., 2020b). Among cryoprotectants, Dimethylsulphoxide (Me_2_SO) and Ethylene glycol (EG) have been shown to be the most effective for vitrification (Almeida-Monteiro et al., 2020a, b).

In addition to the diluent and cryoprotectant, supplements can be added to increase protection to the sperm cell during the process. Various substances present in the cell’s cytoplasm help protect them from external damage, however, the cytoplasm of sperm cells is scarce, making them more vulnerable to adversities of the external environment (Saleh and Agarwal, 2002). Sulfated polysaccharides (SP) are complex anionic polymers that have various biological functions for cells (Anand et al., 2016; Costa et al., 2010), therefore could be used as additional protection to the spermatic cell. Pereira et al. (Pereira et al., 2020) used SP extracted from Nile tilapia skin (*O. niloticus*) to supplement the conventional freezing medium of *Colossoma macropomum* sperm and obtained promising results. Thus, SP extracted from Nile tilapia skin represent a good alternative to supplement the dilution medium necessary for the seminal cryopreservation process in fish.

In this context, there is a rheophilic species native to Northeast Brazil, the *Prochilodus brevis*, with socio-economic and ecological relevance to the region and is threatened by anthropic actions. This species has been used as an experimental model in research aiming at the application of vitrification biotechnology in fish semen Almeida-Monteiro et al.*,* 2020). Such a study found that EG, at a concentration of 30%, is the most suitable cryoprotectant for seminal vitrification of *P. brevis*, however the best diluent for this species unknown. In addition, there are few reports in the literature on the supplementation of the freezing medium for the vitrification process (Figueroa et al., 2013, 2015), and the influence of the frozen volume on the survival of fish sperm subjected to rapid freezing has not yet been tested.

Thus, the present research aimed to determine the most suitable diluent for the vitrification of *P. brevis* sperm, as well as to evaluate the influence of frozen volume and supplementation of the freezing medium with sulfated polysaccharides extracted from Nile tilapia skin on the quality of *P. brevis* sperm undergoing the vitrification process.

## Methods

### Experimental design

The study was approved by the Animal Research Ethics Committee of the State University of Ceará (4876710/2018). The experiment was carried out in December 2019 at the Fish Reproduction Biotechnology Laboratory (LBRP) of the State University of Ceará (UECE) and at the Laboratory of Marine Biochemistry (Biomar) of the Federal University of Ceará (UFC), Fortaleza, Ceará, Brazil. The use of two diluents, Glucose 5% and Fish-Specific Powder Coconut Water (ACP-104; ACP^TM^, ACP Serviços Tecnológicos Ltda, ACP Biotecnologia, Fortaleza, Ceará, Brazil), was evaluated, as well as the freezing medium supplementation with SP extracted from Nile tilapia skin. The storage of samples in two different volumes, 60 μL and 420 μL, was also tested.

### Extraction of sulfated polysaccharides from Nile tilapia skin

The skin of *O. niloticus* was obtained from specimens grown at the Federal University of Ceará, Fortaleza, Ceará, Brazil. The extraction of polysaccharides was performed according to the methodology of Farias et al. (2000) and Salles et al. (2017)

In brief, the skin was initially dehydrated at 45 °C for 6h. Subsequently, 20 g of this dehydrated skin was digested with 10% papain (24 h, 60°C) in 200 mL of 100 mM sodium acetate buffer, containing cysteine and EDTA (both at 5 mM). After incubation, the material was filtered through a nylon net and allowed to precipitate for 72 h at room temperature, with 10 mL of 10% cetylpyridinium chloride (CPC). Following centrifugation (9,560 × g; 20 min, 25°C), the SP obtained were washed with 100 mL of 0.05% CCP, dissolved in 50 mL of a 2M NaCl: ethanol solution (100: 15, v: v) for 20 minutes and precipitated with 100 mL of ethanol (4°C, 72 h). After another centrifugation, the SP were washed twice with 100 mL of 80% ethanol and once again with the same volume of commercial ethanol, then dried in an air circulation oven (60 °C; 6 h). After drying, the material was weighed and stored.

### Experimental animals and gamete collection

We selected 16 males of *P. brevis* (weight: 156.15 ± 26.94 g; length: 20.73 ± 1.48 cm), which were housed in 7100 L fiberglass tanks with constant aeration and fed daily with commercial feed. The fishs were hormonally induced to produce sperm through intracoelomic injections of pituitary carp extract (PCE) administered in two doses (0.4 mg PCE/kg of live weight and 4.0 mg PCE/kg of live weight) with an interval of eight hours between them.

Sixteen hours after the administration of the second hormonal dose, each animal was sedated using a solution based on clove oil (Eugenol; Sigma-Aldrich Brasil Ltda, Rio de Janeiro, RJ, Brazil), in the proportion of 1:10:10000 (Eugenol: absolute alcohol: tank water), and the semen was immediately collected in sterile 3 mL syringes. The samples were kept in a thermal box at 4 ºC until processing. Six *pools* were formed, and single aliquots were allocated to the following spermatic analyses: kinetics (total motility, curvilinear velocity - VCL, average path velocity - VAP and straight-line velocity - VSL), DNA integrity and cytoplasmic membrane integrity.

### Sperm analysis

Sperm kinetics were evaluated using a computer-aided seminal analysis system (CASA - Computer Assisted Sperm Analysis), with the assistance of Sperm Class Analyzer software (SCA, Microptics - Barcelona - Spain, version 3.2), configured for fish. For this purpose, an aliquot of fresh semen (1 μL) and vitrified semen (4 μL) was placed in Makler chambers and activated with 100 μL of NaCl 125 mM (220 mOsm/kg). The evaluated parameters were total motility (%), VCL (μm/s), VSL (μm/s) and VAP (μm/s), with at least 1000 spermatozoa evaluated per analysis.

The analysis of DNA integrity was based on the sperm chromatin fragmentation rate, performed by the SCD (Sperm Chromatin Dispersion) test, following adapted methodology from Fernandez et al. (2005). For this purpose, 0.1 µL of fresh sperm solution and 1 µL of vitrified sperm solution, were diluted in 1.5 mL phosphate-saline buffer (PBS) and kept in a water bath at 37°C until further use. Then, 25 µl of PBS- sperm solution was mixed with 50 µl of low-molecular-weight agarose (Low-Weight Agarose; Sigma-Aldrich, St Louis, MO, USA), and 2 µl aliquots of this mixture were deposited at each of the 10 points on a slide previously prepared with highly purified agarose (NA Agarose; Sigma-Aldrich, St Louis, MO, USA). The slides were covered with coverslips and placed on a cooled metal surface at 4°C for five minutes. Subsequently, the coverslips were removed and the slides were subjected to different solutions: acid solution (hydrochloric acid (HCl) and milliqué water, for seven minutes); lysis solution (sodium chloride (NaCl), sodium dodecyl sulfate (SDS), triton X, ethylene diaminetetraacetic acid (EDTA), β-mercaptoethanol, Tris-HCL solution, and distilled water, for 25 minutes); distilled water (for five minutes); and 70% alcohol, 90% alcohol, and absolute alcohol, respectively (for two minutes each). After the bath procedure, samples were stained with a Panotic kit (RenyLab Chemical and Pharmaceutical Ltda., Barbacena County, MG, Brazil); each slide was dipped in each dye for 10, 20, and 20 seconds, respectively. Finally, the slides were washed in distilled water and dried at room temperature. Two hundred sperm cells were evaluated using a camera-coupled phase contrast optical microscope (200×; Nikon Eclipse 50 i, Tokyo, Japan) to investigate the halo incidence around the sperm head. Cells presenting an external halo indicated sperm chromatin dispersion (intact DNA), while halo-free cells indicated DNA fragmentation.

To evaluate the sperm cytoplasmic membrane integrity, the eosin-nigrosin staining method, adapted from Blom (1950), was employed. One slide per sample was prepared, where a mixture of 5 μL of semen with 10 μL of eosin and 10 μL of nigrosin (ratio 1:2:2 - semen: eosin: nigrosin) was used to creat smears. Under a light microscope (400x), 200 spermatozoa were analyzed. Sperm were considered to have intact membrane when colorless, or with ruptured membrane when colored pink or red.

### Sperm vitrification

One aliquot of each *pool* was diluted (1:9 – semen: freezing medium) in a cryoprotective medium (CM), composed of 30% EG, 4.5% sucrose (0.13 M) and 2% Bovine Serum Albumin (BSA), combined with 5% Glucose or ACP-104 and supplemented or not with 0.5 mg/mL of SP extracted from Nile tilapia skin.

After dilution, vitrification was performed using the droplet method, where in 20 μL of each sample were placed directly into liquid nitrogen, forming solid spheres. These spheres were then transferred to 1.5 mL cryotubes (Corning Incorporated®) previously cooled in liquid nitrogen. In each cryotube, either 3 spheres (60 μL - minimum volume required to perform the analyses) or 21 spheres (420 μL – almost full cryotube) were allocated for each freezing medium, what formed a total of 8 treatments:

T1 - CM + 5% Glicose in 3 spheres;
T2 - CM + 5% Glicose in 21 spheres;
T3 - CM + 5% Glicose + SP in 3 spheres;
T4 - CM + 5% Glicose + SP in 21 spheres;
T5 - CM + ACP-104 in 3 spheres;
T6 - CM + ACP-104 in 21 spheres;
T7 - CM + ACP-104 + SP in 3 spheres;
T8 - CM + ACP-104 + SP in 21 spheres.

This procedure was performed in quadruplicate, and cryotubes were stored in cryogenic cylinder at -196 °C.

After 15 days, the samples were devitrified following the adapted methodology of Merino et al. (2011): a defrosting solution, composed of 2% BSA and 5% Glucose or ACP-104, and kept in a water bath at 37 ºC, was used. Thus, 1 mL (treatments with 3 spheres) or 2 mL (treatment with 21 spheres) of this solution was added to the samples, followed by manual agitation until complete defrosting (approximately five seconds). The samples were then centrifuged at 14.000 rpm (10.000 x g) for 15 minutes using micro-centrifuge (Senova Biotech Co., Ltd, Shanghai, China), to eliminate cryoprotectants. The sediment was resuspended in 100 μL and 200 μL, respectively, of the defrosting solution and, finally, submitted to the same seminal analyses described above.

### Statistical analysis

Initially, the data were subjected to the Shapiro-Wilk and Bartlett tests to verify the normal distribution of residues and homoscedasticity, respectively. When necessary, data underwent a logarithmic transformation to fit the variance analysis (ANOVA), which was performed using SAS software PROC GLM (2002). A completely randomized experimental design was considered, at a 2 x 2 x 2 factorial arrangement (diluent x supplementation with SP x stored volume), as well as the interaction between them. Comparisons between means were made using Student Newman Keuls test (SNK). Data were expressed as mean ± standard error of the means, considering a significance level of 5% (P < 0.05).

## Results

The semen collected from all animals (0,92 ± 0,26 mL) was used to create the six *pools,* which had a mean sperm concentration of 40.02 ± 4.28 x 10^9^ spermatozoa per mL of semen. Concerning kinetic parameters, the mean total motility rate was 96.35 ± 1.62% and the means of VCL, VSL and VAP were 125.24 ± 11.95 μm/s, 68.57 ± 6.37 μm/s and 111.78 ± 12.28 μm/s, respectively. Regarding the other parameters, 92.25 ± 1.97% of spermatozoa presented intact membrane and 95.92 ± 2,20% intact DNA.

Regarding vitrified semen, all variables (diluent, supplementation with SP and stored volume) independently influenced some parameters, and interactions between them were found.

The diluent influenced the parameters of total motility, VAP, membrane and DNA integrity. The stored volume influenced the VSL, membrane and DNA integrity parameters. The presence of SP influenced membrane and DNA integrity. Regarding parameters with interactions between variables, the following stand out: double interaction (diluent-volume) for the parameters of total motility, VAP and membrane integrity; and triple interaction (diluent-volume-SP) for DNA integrity.

**Figure 1:**
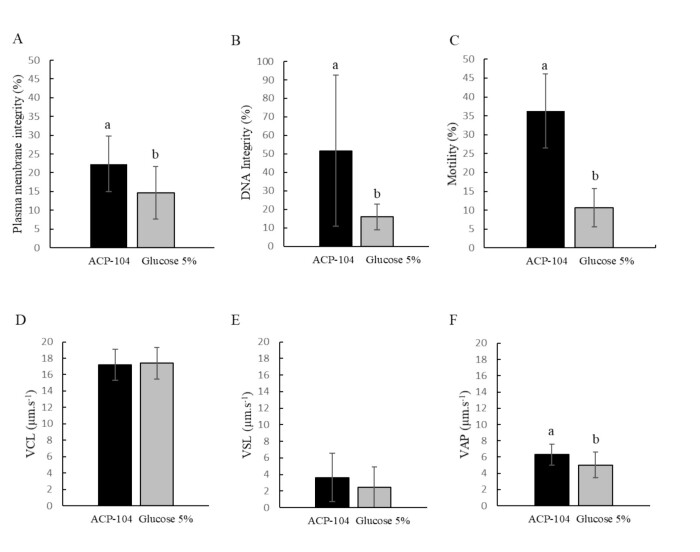
Influence of diluent (Powder Coconut Water - ACP-104 or 5% Glucose) on the quality of *P. brevis* vitrified sperm, regardless of the stored volume and supplementation with Sulfated Polysaccharides. (A) Plasma membrane integrity; (B) DNA integrity; (C) Total motility; (D) Curvilinear velocity - VCL; (E) Straight line velocity - VSL and (F) Average path velocity - VAP. a'b indicate statistical difference between diluents, regardless of the stored volume and the use of sulfated polysaccharides. (P < 0.05).

The best (P < 0.05) results for total motility rate (36.27 ± 2.00%), VAP (6.31 ± 0.28 μm/s), membrane (22.35 ± 1.51%) and DNA (51.70 ± 8.34%) integrity were achievied when ACP-104 was used as a diluent for the *P. brevis* sperm vitrification, regardless of the stored volume and the supplementation with PS. No significant differences for VCL and VSL were found (Figure 1).

**Figure 2:**
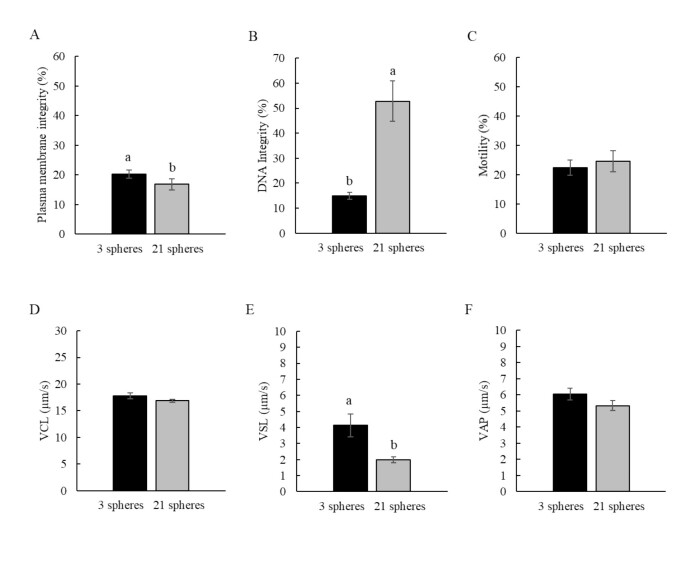
Influence of stored volume (3 spheres - 60 μL or 21 spheres - 420 μL) on the quality of *P. brevis* vitrified sperm, regardless of the diluent used and supplementation with Sulfated Polysaccharides. (A) Plasma membrane integrity; (B) DNA integrity; (C) Total motility; (D) Curvilinear velocity - VCL; (E) Straight line velocity - VSL and (F) Average path velocity - VAP. a'b indicate statistical difference between stored volumes, regardless of the diluent and the use of sulfated polysaccharides. (P < 0.05).

Considering the stored volume, irrespective of diluent and supplementation with SP, the best results (P < 0.05) of VSL (4.14 ± 0.71 μm/s) and membrane integrity (20.26 ± 1.32%) were obtained when vitrified semen was stored in the smaller volume (60 μL). However, the highest (P < 0.05) DNA integrity rates (52.75 ± 8.13%) were obtained when using the largest stored volume (420 μL), and no significant differences for the other kinetic parameters were observed (Figure 2).

**Figure 3:**
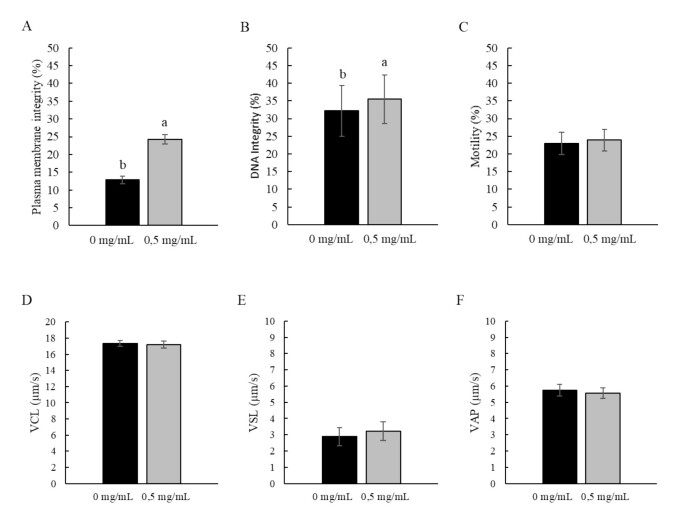
Influence of the supplementation of the cryodiluent medium with sulfated polysaccharides extracted from the Nile tilapia skin (0 and 0.5 mg/mL) on the quality of the *P. brevis* vitrified sperm, regardless of the stored volume and the diluent used. (A) Plasma membrane integrity; (B) DNA integrity; (C) Total motility; (D) Curvilinear velocity - VCL; (E) Straight line velocity - VSL and (F) Average path velocity - VAP. a'b indicates statistical difference between the use or not of sulfated polysaccharides, regardless of the diluent and the stored volume. (P < 0.05).

Conversely, cryodiluent medium supplementation with SP, extracted from Nile tilapia skin, was beneficial (P < 0.05) for membrane (24.22 ± 1.31%) and DNA (35.50 ± 6.88%) integrity parameters, regardless of the diluent used and the stored volume, and no influence on the other evaluated parameters was noted (Figure 3).

There was interaction (P < 0.05) between diluents and stored volume for total motility, VAP and membrane integrity parameters. When comparing the diluents within the same stored volume, ACP-104 showed a higher motility rate (P < 0.05) than 5% Glucose for both volumes. However, when comparing the different volumes within the same diluent, no significant differences were found (Table 1).

**Table 1:** Motility rate, Average Pathway Velocity (VAP) and Membrane Integrity of *Prochilodus brevis* sperm vitrified with 5% Glucose or Powder Coconut Water (ACP-104) diluents and stored in different volumes (60 μL – 3 spheres or 420 μL- 21 spheres).

	**Glucose 5%**		**ACP-104**	
	**3 spheres**	**21 spheres**	**3 spheres**	**21 spheres**
**Motility (%)**	13.16 ± 1.78^Ba^	8.02 ± 0.96^Ba^	31.39 ± 3.24^Aa^	40.23 ± 1.17^Aa^
**VAP (µm/s)**	6.07 ± 0.55^Aa^	4.16 ± 0.21^Bb^	6.05 ± 0.51^Aa^	6.51 ± 0.31^Aa^
**Membrane Integrity (%)**	18.75 ± 1.84^Aa^	10.71 ± 1.49^Bb^	21.78 ± 1.86^Aa^	22.91 ± 2.45^Aa^

A'B indicate statistical difference between diluents in the same volume. a'b indicate statistical difference between volumes in the same diluent. (P < 0.05).

The VAP and membrane integrity parameters follow a similar pattern: when comparing different diluents within the same volume, ACP-104 was superior (P< 0.05) to 5% Glucose when the semen was stored in the larger volume. However, no significant differences between the diluents were found when using the smaller volume. When considering different stored volumes within the same diluent, 5% Glucose showed better results (P < 0.05) when using the smaller volume, while for ACP-104 there was no significant difference between the stored volumes (Table 1).

There was interaction (P < 0.05) between diluents, stored volumes and supplementation with SP (triple interaction) for DNA integrity parameter (Table 2).

**Table 2:** DNA integrity of the *Prochilodus brevis* sperm vitrified with different diluents (5% Glucose or Powder Coconut Water - ACP-104), supplemented or not with Sulfated Polysaccharides extracted from Nile tilapia skin (SP) and stored in different volumes (60 μL - 3 spheres or 420 μL - 21 spheres).

**DNA Integrity (%)**			
**Stored volume**	**SP from Nile** **tilapia skin**	**Glucose 5%**	**ACP-104**
3 spheres	0 mg/mL	10.00 ± 0.58^Aa^	14.33 ± 1.28^Ba^
	0.5 mg/mL	25.83 ± 0.91^Aa^*	9.50 ± 0.96 ^Bb^
21 spheres	0 mg/mL	12.67 ± 1.67^Ab^	91.67 ± 1.26^Aa^
	0.5 mg/mL	15.33 ± 2.29^Bb^	91.33 ± 2.36^Aa^

A'B indicate statistical difference between the stored volumes, within the same diluent and the same SP concentration. a'b indicate statistical difference between diluents, within the same stored volume and the same SP concentration. *indicates statistical difference between SP concentrations, within the same stored volume and the same diluent. (P < 0,05)

When considering different stored volumes, using the same diluent and the same SP concentration, 5% Glucose presented better rates (P < 0.05) of DNA integrity when using the smaller volume, for the medium supplemented with SP. However, there was no significant difference between the volumes for the non-supplemented medium. For ACP-104, the best results (P < 0.05) were obtained when vitrified semen was stored in the larger volume, both for the supplemented and the non-supplemented with SP medium (Table 2).

Additionally, concerning the different diluents, using the same stored volume and the same SP concentration, 5% Glucose was superior (P < 0.05) to ACP-104 when the semen was supplemented with SP and stored in the smaller volume, however, no significant difference between the diluents were found when semen without supplementation was stored in the smaller volume. When storing the semen in the larger volume, the ACP-104 medium, supplemented or not with SP, was significantly superior (P < 0.05) to 5% glucose (Table 2).

Regarding the supplementation of the cryodiluent medium, using the same diluent and the same stored volume, the use of SP improved (P < 0.05) the *P. brevis* spermatozoa DNA integrity rate when using 5% Glucose and the smaller volume. This supplement had no influence on the other diluents and volumes (Table 2).

## Discussion

The present research investigated the efficacy of the two most commonly used diluents for *P. brevis* cryopreservation techniques, 5% Glucose and Fish-Specific Powder Coconut Water (ACP-104). A previous study (Almeida-Monteiro et al., 2020b) had determined the optimal concentration of various cryoprotectants for *P. brevis* sperm vitrification, identifying 30% EG, combination with 4.5% Sucrose and 2% Bovine Serum Albumin, as the most effective cryoprotectant medium. However, that study did not establish the best diluent for the semen vitrification process. This gap was addressed in the present study, which identified ACP-104 as the superior diluent for most of the evaluated parameters. ACP-104 yielded a motility rate exceeding 35%, membrane integrity suparssing 20% and DNA integrity exceeding 50%.

Moreover, we assessed the impact of the semen stored volume after the vitrification process. It is well-documented that achieving the vitreous state, necessitates freezing the sample in small volumes (Slabbert et al., 2015; Xin et al., 2017). However, small frozen volumes can complicate captive production process, as a minimum spermatozoa to oocyte ratio (inseminating dose) is required for in vitro fertilization. For *P. brevis*, the recommended inseminating dose is approximately 928,410.29 spermatozoa per oocyte (Oliveira-Araújo et al., 2020), requiring about 0.23 μL of vitrified sperm to attain this concentration. Considering an average of 1300 oocytes per gram of spawn, and approximately 28.4 grams (Oliveira-Araujo et al., 2020), approximately 8.5 mL of vitrified semen would be needed to fertilize all oocytes of a single *P. brevis* female. Therefore, our aim was to determine whether storing samples in larger volumes would compromise vitrified spermatozoa, given that freezing was conducted using the droplet method (freezing in small volumes), wich should not interfere on the proper rapid freezing process.

Another critical consideration in the vitrification process is recrystallization, the formation of ice crystals during sample defrosting. Research indicates that, when employing high freezing rates, correspondingly high defrosting rates should be applied (Cuevas-Uribe et al., 2011; Mazur and Seki, 2011; Thirumala et al., 2005). This suggests that if freezing occurs rapidly, defrosting should also be rapid to minimize recrystallization. The defrosting method utilized in our study, adapted from Merino et al. (Merino et al., 2011), facillitated swift defrosting rates, effectively reducing recrystallization in both stored volumes. This is evidenced by the favorable DNA integrity results obtained when storing samples vitrified with ACP-104 in the larger volume.

Futhermore, for both diluents, there was no discernible impact of stored volume on motility rates, a parameter widely regarded as crucial for sperm quality assessment (Solis-Murgas et al., 2014). Therefore, it can be inferred that storing vitrified samples in larger volumes may offer significant advantages for captive production processes.

We also investigated the supplementation of the cryodiluent medium with SP extracted from Nile tilapia skin to enhance the protection of sperm cells during the semen freezing and defrosting processes. These substances play vital roles for cells (Costa et al., 2010; Anand et al., 2016). In our study, the addition of SP had a positive impact the membrane and DNA integrity rates of vitrified *P. brevis* sperm. Similarly, Pereira et al. (2020) achieved favorable membrane integrity rates of *C. macropomum* sperm, subjected to conventional cryopreservation, by supplementing the freezing medium with SP extracted from Nile tilapia skin, at concentrations ranging from 0.1 to 1 mg/mL.

Sulfated polysaccharides are polymers composed of repetitive units of sugars containing sulfate radicals, imparting negative charges. These structural characteristics endow them with various biological functions, including anti-inflammatory, anticoagulant and antioxidant properties (Brito et al., 2008; Costa et al., 2010;Anand et al., 2016) In vertebrate animals, these polysaccharides exist in the form of glycosaminoglycans (GAGs), among which dermatan sulfate is one of the most abundant in the skin of several fish species (Sakai et al., 2003; Souza et al., 2007; Mansour et al., 2009). Hence, researchers have turned to Nile tilapia (*O. niloticus*) skin as a natural source of SP (Salles et al., 2017) given its status as the world’s most cultivated species (FAO, 2020), leading to significant production of residues that purposed.

Research indicates that Nile tilapia skin, in the form of gelatin, contains high concentrations of compounds with antioxidant properties (Zhang et al., 2012). Additionally, recent unpublished studies have confirmed the presence of various GAGs in Nile tilapia skin and have demonstrated the antioxidant capacity of these SP. Cryopreservation is a stressful process for cells, rendering them more susceptible to attack by reactive oxygen species (Shaliutina-Kolešová et al., 2015). Moreover, sperm plasma membranes are rich in polyunsaturated fatty acids, which are highly susceptible to lipid peroxidation, and sperm cells are deficient in cytoplasm, a source of antioxidant substances (Saleh and Agarwal, 2002; Wathes et al., 2007).

Another crucial aspect is that SP have the ability to bind to proteins from both the membrane and extracellular matrix to form proteoglycans. These proteoglycans play a vital role in regulating numerous signaling pathways and maintaining osmotic pressure, in addition to acting as excellent water retainers (Iozzo and Schaefer, 2015). Therefore, it is hypothesized that the potencial antioxidant action of SP, combined with their ability to form proteoglycans, may have contributed to the protection of *P. brevis* sperm membranes, including both the cytoplasmic membrane and the cariotheca. This hypothesis is supported by the observation that the best results for membrane and DNA integrity were obtained when the cryodiluent medium was supplemented with 0.5 mg/mL of SP.

An interesting fact to note in our study is that there seemed to be a better action of SP when using the diluent 5% Glucose. This can be explained by the simplicity of this diluent, while ACP-104 has a much more complex composition. ACP^®^ is basically composed of carbohydrates, amino acids, vitamins, minerals, saturated fatty acids, growth factors and even phytohormones, such as 3-indole-acetic acid (AIA), responsible for promoting cell growth (Nunes and Combarnous, 1995; Carvalho et al., 2006). This diluent constitution is made as close as possible to the one of in natura semen of the intended animal variety, including a similar osmolarity, and possibly already having protective substances. Thus, we can deduce that these characteristics are the reason to not observe a more significant action of SP when added to the more complex diluent.

Regarding the interaction between diluent and stored volume, it is possible to observe that the diluents follow an opposite pattern: when 5% Glucose was used, the best results were obtained with the smaller stored volume, while ACP-104 was more efficient, numerically, when stored in a larger volume. This fact emphasizes the importance of testing different diluents, since there may be interaction between factors that cause each one to behave in a different way (Salmito-Vanderley et al., 2012). Then, it is necessary to evaluate the advantages and disadvantages to choose the most appropriate diluent.

We can point out that the assessment of DNA integrity is of great importance to verify the quality of cryopreserved sperm, because this parameter directly influences the survival and development of embryos (Figueroa et al., 2018), since there is a strong correlation between damage to genetic material and the incidence of mutations (Twigg et al., 1998). Fernández-Díez et al. (2015) report that the oocyte has some ability to repair damage to genetic material and generate viable offspring. They also suggest the use of semen with at least 90% of intact DNA sperm for assisted fertilization of fish. In the present study, this result was only achieved when the *P. brevis* sperm was vitrified with ACP-104 and stored in the larger volume, what emphasizes once again the efficiency of this diluent, as well as the feasibility of storing the samples in larger quantities.

In view of the above, the results elucidated in the present study indicate that vitrification is a promising biotechnique for *P. brevis* sperm technology and it can be applied with relative success. However, further studies are needed to better explore the application of SP extracted from Nile tilapia skin. That might be done through tests using other concentrations, as well as other forms of this product, such as gelatin, in order to evaluate the antioxidant effects of this substance. It is also suggested to perform new evaluations of sperm quality after defrosting, such as mitochondrial and antioxidant activity tests, and to verify the fertilization and hatching rates using vitrified sperm.

## Conclusion

In conclusion, Fish-Specific Powder Coconut Water (ACP-104), associated with 30% Ethylene glycol, 4.5% sucrose and 2% BSA, supplemented with 0.5 mg/mL of sulfated polysaccharides extracted from Nile tilapia skin, composes the best cryodiluent for *P. brevis* sperm vitrification, and samples can be stored in large volumes with no prejudice to the sperm quality.

## References

[B001] Almeida-Monteiro PS, Oliveira-Araújo MS, Castelo Branco JS, Salmito-Vanderley CSB (2020). Vitrificação de sêmen de peixes teleósteos. Rev Bras Reprod Anim.

[B002] Almeida-Monteiro PS, Pinheiro RRR, Oliveira-Araújo MS, Sales YS, Nascimento RV, Nunes LT, Pereira VA, Montenegro AR, Melo-Maciel MAP, Salmito-Vanderley CSB (2020). Sperm vitrification of *Prochilodus brevis* using Powder Coconut Water (ACP-104) in association with different cryoprotectant concentrations. Aquacult Res.

[B003] Anand N, Rachel D, Thangaraju N, Anantharaman P (2016). Potential of marine algae (sea weeds) as source of medicinally important compounds. Plant Genet Resour.

[B004] Bakhach J (2009). The cryopreservation of composite tissues: principles and recent advancement on cryopreservation of different type of tissues. Organogenesis.

[B005] Blom E (1950). A one-minute live-dead sperm stain by means of eosin-nigrosin. Fertil Steril.

[B006] Brito AS, Arimateia DS, Souza LR, Lima MA, Santos VO, Medeiros VP, Ferreiro PA, Silva RA, Ferreira CV, Justo GZ, Leite EL, Andrade GPV, Oliveira FW, Nader HB, Chavante SF (2008). Anti-inflammatory properties of a heparin-like glycosaminoglycan with reduced anti-coagulant activity isolated from a marine shrimp. Bioorg Med Chem.

[B007] Carvalho JM, Maia GA, Sousa PHM, Maia GA (2006). Water of coconut: nutritional and functional properties and processing. Semina: Ciênc Agrár.

[B008] Costa LS, Fidelis GP, Cordeiro SL, Oliveira RM, Sabry DA, Câmara RBG, Nobre LTDB, Costa MSSP, Almeida-Lima J, Farias EHC, Leite EL, Rocha HAO (2010). Biological activities of sulfated polysaccharides from tropical seaweeds. Biomed Pharmacother.

[B009] Cuevas-Uribe R, Yang H, Daly J, Savage MG, Walter RB, Tiersch TR (2011). Production of F1 offspring with vitrified sperm from a live-bearing fish, the green swordtail Xiphophorus hellerii. Zebrafish.

[B010] Farias WRL, Valente AP, Pereira MS, Mourão PAS (2000). Structure and anticoagulant activity of sulfated galactans isolation of a unique sulfated galactan from the red algae *Botryocladia occidentalis* and comparison of its anticoagulant action with that of sulfated galactans from invertebrates. J Biol Chem.

[B011] Fernandez JL, Muriel L, Goyanes V, Segrelles E, Gosálvez  J, Enciso M, LaFromboise  M, De Jonge C (2005). Simple determination of human sperm DNA fragmentation with an improved sperm chromatin dispersion test. Fertil Steril.

[B012] Fernández-Díez C, González-Rojo S, Montfort J, Le Cam A, Bobe J, Robles V, Pérez-Cerezales S, Herráez MP (2015). Inhibition of zygotic DNA repair: transcriptome analysis of the offspring in trout (*Oncorhynchus mykiss*). Reproduc..

[B013] Figueroa E, Farias JG, Lee-Estevez M, Valdebenito I, Risopatrón J, Magnotti C, Romero J, Watanabe I, Oliveira RPS (2018). Sperm cryopreservation with supplementation of α-tocopherol and ascorbic acid in freezing media increase sperm function and fertility rate in Atlantic salmon (*Salmo salar*). Aquaculture.

[B014] Figueroa E, Merino O, Risopatron J, Isachenko V, Sanchez R, Effer B, Isachenko E, Farias JG, Valdebenito I (2015). Effect of seminal plasma on Atlantic salmon (Salmo salar) sperm vitrification. Theriogenology.

[B015] Figueroa E, Risopatrón J, Sánchez R, Isachenko E, Merino O, Isachenko V, Valdebenito I (2013). Sperm vitrification of sex-reversed rainbow trout (Oncorhynchus mykiss): effect of seminal plasma on physiological parameters. Aquaculture.

[B016] FAO (2020). The State of World Fisheries and Aquaculture.

[B017] Iozzo RV, Schaefer L (2015). Proteoglycan form and function: a comprehensive nomenclatureof proteoglycans. Matrix Biol.

[B018] Kása E, Bernath G, Kollar T, Zarski D, Lujic J, Marinovic Z, Bokor Z, Hegyi A, Urbanyi B, Vilchez MC, Morini M, Peñaranda DS, Pérez L, Asturiano JF, Horvath A (2017). Development of sperm vitrification protocols for freshwater fish (Eurasian perch, *Perca fluviatilis*) and marine fish (European eel, *Anguilla anguilla*). Gen Comp Endocrinol.

[B019] Mansour MB, Majdoub H, Bataille I, Roudesli M, Hassine M, Ajzenberg N, Chaubet F, Maarouf RM (2009). Polysaccharides from the skin of the ray Raja radula. Partial characterization and anticoagulant activity. Thromb Res.

[B020] Mazur P, Seki S (2011). Survival of mouse oocytes after being cooled in a vitrification solution to - 196 C at 95 to 70,000 C/min and warmed at 610 to 118,000 C/min: a new paradigm for cryopreservation by vitrification. Cryobiology.

[B021] Merino O, Risopatrón J, Sánchez R, Isachenko E, Figueroa E, Valdebenito I, Isachenko V (2011). Fish (Oncorhynchus mykiss) spermatozoa cryoprotectant-free vitrification: stability of mitochondrion as criterion of effectiveness. Anim Reprod Sci.

[B022] Naik BR, Rao BS, Vagdevi R, Gnanprakash M, Amarnath D, Rao VH (2005). Conventional slow freezing, vitrification and open pulled straw (OPS) vitrification of rabbit embryos. Anim Reprod Sci.

[B023] Nunes JF, Combarnous Y (1995). Utilização da água de coco como diluidor do sêmen dos mamíferos domésticos..

[B024] Oliveira-Araújo MS, Lopes JT, Nunes LT, Almeida-Monteiro PS, Nascimento RV, Pereira VA, Ferreira YM, Neres WR, Leite-Castro LV, Montenegro AR, Salmito-Vanderley CSB (2020). Determination of the ideal volume of activating solution and the optimal ratio of spermatozoa per oocyte for Prochilodus brevis fertilization. Zygote.

[B025] Pereira VA, Alencar DB, Araújo IWF, Rodrigues JAG, Lopes JT, Nunes LT, Ferreira YM, Lobato JS, Montenegro AR, Salmito-Vanderley CSB (2020). Supplementation of cryodiluent media with seaweed or Nile tilapia skin sulfated polysaccharides for freezing of Colossoma macropomum (Characiformes: Serrasalmidae) sêmen. Aquaculture.

[B026] Rubinsky B (2003). Principles of low temperature cell preservation. Heart Fail Rev.

[B027] Sakai S, Kim WS, Lee IS, Kim YS, Nakamura A, Toida T, Imanari T (2003). Purification and characterization of dermatan sulfate from the skin of the eel, *Anguilla japônica*. Carbohydr Res.

[B028] Saleh RA, Agarwal A (2002). Oxidative stress and male infertility: from research bench to clinical practice. J Androl.

[B029] Salles TC, Rodrigues JAG, Barcellos PG, Amaral GF, Araújo IWF, Souza Mourão PA (2017). Inhibition of thrombin generation by dermatan sulfate isolated from the skin of Oreochromis niloticus. Agraria.

[B030] Salmito-Vanderley CSB, Vieira MJAF, Leite LV, Oliveira FCE, Linhares FRA, Salgueiro CCM, Nunes JF (2012). Meios de congelação para conservação de sêmen de peixes da família Characidae. Ciênc Anim.

[B031] Shaliutina-Kolešová A, Cosson J, Lebeda I, Gazo I, Shaliutina O, Dzyuba B, Linhart O (2015). The influence of cryoprotectants on sturgeon (Acipenser ruthenus) sperm quality, DNA integrity, antioxidant responses, and resistance to oxidative stress. Anim Reprod Sci.

[B032] Slabbert M, Du Plessis SS, Huyser C (2015). Large volume cryoprotectant-free vitrification: an alternative to conventional cryopreservation for human spermatozoa. Andrologia.

[B033] Solis-Murgas LD, Oliveira Felizardo V, Souza Andrade E, Ferreira MR, Jesus Paula DA, Carvalho AFS, Solis-Murgas LD, Souza G (2014). Recent Advances in Cryopreservation..

[B034] Souza MLS, Dellias JMM, Melo FR, Silva LCF (2007). Structural composition and anticoagulant activity of dermatan sulfate from the skin of the electric eel, Electrophorus electricus (L). Comp Biochem Physiol B Biochem Mol Biol.

[B035] Thirumala S, Huang C, Dong Q, Tiersch TR, Devireddy RV (2005). A theoretically estimated optimal cooling rate for the cryopreservation of sperm cells from a live-bearing fish, the green swordtail Xiphophorus helleri. Theriogenology.

[B036] Twigg JP, Irving DS, Aitken RJ (1998). Oxidative damage to DNA in human spermatozoa does not preclude pronucleus formation at intracytoplasmatic sperm injection. Hum Reprod.

[B037] Varela-Junior A, Goularte KL, Alves JP, Pereira FA, Silva EF, Cardoso TF, Jardim RD, Streit DP, Corcini CD (2015). Methods of cryopreservation of Tambaqui semen, Colossoma macropomum. Anim Reprod Sci.

[B038] Wathes DC, Abayasekara DRE, Aitken RJ (2007). Polyunsaturated fatty acids in male and female reproduction. Biol Reprod.

[B039] Xin M, Siddique MAM, Dzyuba B, Cuevas-Uribe R, Shaliutina-Kolešová A, Linhart O (2017). Progress and challenges of fish sperm vitrification: a mini review. Theriogenology.

[B040] Zhang Y, Duan X, Zhuang Y (2012). Purification and characterization of novel antioxidante peptides from enzymatic hydrolysates of tilapia (Oreochromis niloticus) skin gelatin. Peptides.

